# A Decision-Making Approach Based on Score Matrix for Pythagorean Fuzzy Soft Set

**DOI:** 10.1155/2021/5447422

**Published:** 2021-10-26

**Authors:** Imran Siddique, Rana Muhammad Zulqarnain, Rifaqat Ali, Alhanouf Alburaikan, Aiyared Iampan, Hamiden Abd El-Wahed Khalifa

**Affiliations:** ^1^Department of Mathematics, School of Science, University of Management and Technology, Lahore 54770, Pakistan; ^2^Department of Mathematics, School of Science, University of Management and Technology, Lahore, Sialkot Campus, Pakistan; ^3^Department of Mathematics, College of Science and Arts, King Khalid University, Muhayil 61413, Abha, Saudi Arabia; ^4^Department of Mathematics, College of Arts and Sciences, Al-Badaya, Qassim University, Buraydah, Saudi Arabia; ^5^Department of Mathematics, School of Science, University of Phayao, Mae Ka, Mueang, Phayao 56000, Thailand; ^6^Operations Research Department, Faculty of Graduate Studies for Statistical Research, Cairo University, Giza, Egypt

## Abstract

Pythagorean fuzzy soft set (PFSS) is the most powerful and effective extension of Pythagorean fuzzy sets (PFS) which deals with the parametrized values of the alternatives. It is also a generalization of intuitionistic fuzzy soft set (IFSS) which provides us better and precise information in the decision-making process comparative to IFSS. The core objective of this work is to construct some algebraic operations for PFSS such as OR-operation, AND-operation, and necessity and possibility operations. Furthermore, some fundamental properties have been established for PFSS utilizing the developed operations. Moreover, a decision-making technique has been offered for PFSS based on a score matrix. To demonstrate the validity of the proposed approach, a numerical example has been presented. Finally, to ensure the practicality of the established approach, a comprehensive comparative analysis has been presented. The obtained results show that our developed approach is most effective and delivers better information comparative to prevailing techniques.

## 1. Introduction

Zadeh [1] introduced the notion of the fuzzy set (FS), which assigns to each object a membership value ranging between zero and one. Generally, decision-makers consider a membership and nonmembership value in the decision-making procedure which cannot be handled by FS. Atanassov [2] generalized the concept of the FS and presented the concept of intuitionistic fuzzy set (IFS) to handle the aforesaid limitation. Atanassov and Gargov [3] extended the idea of IFS and developed the theory of interval-valued fuzzy set (IVFS). Atanassov [2] presented some results for IFS and developed two novel operators for IFS and studied their basic properties. Kaur and Garg [4] developed several aggregation operators (AOs) under the environment of complex IFS and discussed their properties. De et al. [5] expressed dilation, normalization, and concentration of IFS. These concepts are valuable while dealing with different linguistic hedges involved in the complications under the IFS environment. Deschrijver and Kerre [6] defined the relationship between several other extensions of FS and IFS and also discuss the inter-relationship between IFS, soft set, rough set, fuzzy rough set, probabilistic set, L-fuzzy set, and type 2 fuzzy set. Xu [7] defined a new methodology for deriving the correlation coefficient of IFS, which has several benefits over current approaches. He also extended the concept of interval-valued IFS theory and utilized in medical diagnosis. Zulqarnain et al. [8] established the correlation coefficient for interval-valued IFSS and utilized their developed correlation coefficient for the construction of the TOPSIS approach. Zulqarnain and Dayan [9] utilized the intuitionistic fuzzy TOPSIS for the selection of an auto company.

But the existing IFS has some limitations such that the sum of membership grade (MG) and nonmembership grade (NMG) cannot exceed 1. For example, on the condition that we have MG = 0.7 and NMG = 0.7, then clearly, we have MG+NMG > 1 and we can see that this is not handled by the IFS because IFS only deal with the situations, where MG+NMG ≤ 1. Yager [10] protracted the idea of IFS to Pythagorean fuzzy set (PFS) by upgrading the condition MG+NMG ≤ 1 to MG^2^ + NMG^2^ ≤ 1. As a generalized set of IFS, PFS has a close relationship with IFS. The PFS accurately access the uncertain facts than IFS. Muhammad Zulqarnain et al. [11] utilized the intuitionistic fuzzy soft matrices for disease diagnosis. Xu and Yager [12] developed the geometric aggregation operators for IFS. Wei et al. [13] planned several operators for picture fuzzy sets and offered a multiattribute group decision-making (MAGDM) approach. Wang and Li [14] proposed Bonferroni mean AOs for PFS and constructed the multiattribute decision-making (MADM) approach based on their developed operators. Ma and Xu [15] established some novel aggregation operators for PFS and established the multicriteria decision-making approach based on developed operators. Garg et al. [16] extended the TOPSIS method to solve MADM problems under hesitant fuzzy information. Peng and Yang [17] discussed some desirable operations and properties for PFS. Wang et al. [18] introduced the novel dice similarity measures for PFS and developed a MAGDM approach based on their proposed similarity measures. Wang et al. [19] presented interactive Hamacher power AOs for Pythagorean fuzzy numbers and developed a decision-making methodology based on their developed operators. Muhammad Zulqarnain et al. [20] extended the notion of the IFSS to an intuitionistic fuzzy hypersoft set and introduced the TOPSIS approach based on the correlation coefficient. Garg and Arora [21] proposed the generalized AOs for the intuitionistic fuzzy soft set. Zhang and Xu [22] extended the TOPSIS method for PFS and used it for the multicriteria decision-making (MCDM) problem.

The concept of soft set (SS) was established by Molodtsov [23], which deals with parametrized values of the alternative. Maji et al. [24] investigated the SS views on decision-making (DM) issues and defined some important concepts for SS with their properties. Chen et al. [25] developed parameterization reduction of SS with its application. Maji et al. [26] offered the notion of the fuzzy soft set (FSS) by merging two existing notions FS and SS. Kong et al. [27] established a theoretic decision-making approach for FSS theory. Maji et al. [28] prolonged the concept of IFSS, which is a generalization of FSS, and defined new operations on IFSS. Rajarajeswari and Dhanalakshmi [29] developed an intuitionistic fuzzy soft matrix (IFSM) and also describe their application on IFSM. Many researchers expanded SS theory by utilizing the fundamental definition of FSS. Peng et al. [30] protracted the idea of IFSS to PFSS by upgrading the condition MG+NMG ≤ 1 to MG^2^ + NMG^2^ ≤ 1. As a generalized set of IFSS, PFSS has a close relationship with IFSS. Athira et al. [31] proposed the entropy measure based on Hamming distance and Euclidean distance of PFSS. Athira et al. [32] also introduced the distance-based entropy measures and developed a decision-making methodology to solve decision-making complications. Naeem et al. [33] constructed the TOPSIS and VIKOR approaches under linguistic PFSS environs and proposed some fundamental operations with their properties. Riaz et al. [34] extended the notion of PFSS to m-polar PFSS and presented the TOPSIS method for m-polar PFSS to solve multicriteria group decision-making (MCGDM) issues. Riaz et al. [35] proposed the similarity measures for PFSS and established a decision-making approach based on developed similarity measures. Zulqarnain et al. [36] developed the AOs for PFSS and proposed a decision-making methodology based on their established operators. Zulqarnain et al. [37] extended the TOPSIS technique for PFSS based on CC and utilized their established technique for MADM complications. Zulqarnain et al. [38] proposed the interactive AOs for PFSS and constructed an MCDM approach using their developed interactive AOs. It has been observed that fuzzy numbers can only measure uncertainty and intuitionistic fuzzy numbers can measure true and false membership values such as the sum of true and false membership values must be less than 1. But, in our developed methodology, we can measure the values of truth and false membership by modifying the intuitionistic fuzzy numbers condition such as the sum of the square of true and false values must be less than or equal to 1. The main objective of this paper is to develop some logical operators with their properties for PFSS. The rest of this research is ordered as follows: some basic concepts have been discussed in Section 2. In Section 3, we defined some logical operators for PFSS with their fundamental properties. We developed decision making based on the Pythagorean fuzzy soft matrix in Section 4. In Section 5, a brief comparison has been presented with some existing methodologies. In Section 6, the conclusion is given.

## 2. Preliminaries

In this section, we will present several fundamental definitions which help us to develop the construction of the following work such as SS, PFS, IFSS, and PFSS.

**Definition 1 (see [23]).**

Let *X* be a universal set and *ℕ*={*P*_1_, *P*_2_, *P*_3_,…, *P*_*m*_} be the set of attributes, then a pair (Ω, *ℕ*) is called a SS over *X* where Ω : ℕ⟶*K*^*X*^ is a mapping and *K*^*X*^ is known as a collection of all subsets of universal set *X*.

**Definition 2 (see [28]).**

Let *X* be a universal set and ℕ be set of attributes, then a pair (Ω, *ℕ*) is called an IFSS over *X* where Ω : ℕ⟶*IK*^*X*^ is a mapping and *IK*^*X*^ is known as a collection of all IFS subsets of universal set *X*.

(1) $$\begin{array}{c} \left(\Omega ,A\right)=\left\{P,\left({}^{\prime }{ϒ}_{A}\left(P\right),\vartheta_{A}\left(P\right)\right)\;|\;P\in A\right\}, \end{array}$$

where ′ϒ_*A*_(*P*), *ϑ*_*A*_(*P*) : *A*⟶[0,1] are of membership grade and nonmembership functions respectively with 0 ≤ ′ϒ_*A*_(*P*)+*ϑ*_*A*_(*P*) ≤ 1 and *A* ⊂ ℕ.

**Definition 3 (see [8]).**

Let *X* be a collection of objects, then a PFS *A* over *X* is defined as

(2) $$\begin{array}{c} A=\left\{\left(P,{}^{\prime }{ϒ}_{A}\left(P\right),\vartheta_{A}\left(P\right)\right)|P\in X\right\}, \end{array}$$

where ′ϒ_*A*_(*P*), *ϑ*_*A*_(*P*) : *X*⟶[0,1] are membership and nonmembership grade functions, respectively. Furthermore, 0 ≤ ′ϒ_*A*_(*P*)^2^+*ϑ*_*A*_(*P*)^2^ ≤ 1 and *I*=1 − ′ϒ(*P*)^2^ − *ϑ*_*A*_(*P*)^2^ is called degree of indeterminacy.

We can see from the above definitions that the only difference is in the conditions, i.e., in IFS, we deal with the condition 0 ≤ ϒ_*A*_(*P*)+*ϑ*_*A*_(*P*) ≤ 1 and *I*=1 − ′ϒ_*A*_(*P*) − *ϑ*_*A*_(*P*), whereas in PFS, we have condition 0 ≤ ′ϒ_*A*_(*P*)^2^+*ϑ*_*A*_(*P*)^2^ ≤ 1 and *I*=1 − ′ϒ_*A*_(*P*)^2^ − *ϑ*_*A*_(*P*)^2^. We can say that a PFS is the general case of IFS.

**Definition 4 (see [30]).**

Let *X* be a universal set and ℕ be set of attributes, then a pair (Ω, ℕ) is called a PFSS over *X* where Ω : ℕ⟶*℘K*^*X*^ is a mapping and *℘K*^*X*^ is known as the collection of all PFS subsets of universal set *X*.

(3) $$\begin{array}{c} \left(\Omega ,A\right)=\left\{P,\left({}^{\prime }{ϒ}_{A}\left(P\right),\vartheta_{A}\left(P\right)\right)\;|\;P\in A\right\}, \end{array}$$

where ′ϒ_*A*_(*P*), *ϑ*_*A*_(*P*) : *A*⟶[0,1] are of membership grade and nonmembership functions respectively with 0 ≤ ′ϒ_*A*_(*P*)^2^+*ϑ*_*A*_(*P*)^2^ ≤ 1, degree of indeterminacy $ℑ=\sqrt{1-{}^{\prime }{ϒ}_{A}{\left(P\right)}^{2}-\vartheta_{A}{\left(P\right)}^{2}}$, and *A* ⊂ ℕ.

For the sake of readers' convenience, we express the PFSN as ℋ_*ij*_=〈′ϒ_*ij*_, *ϑ*_*ij*_〉. For calculating the ranking of alternatives, Zulqarnain et al. [36] introduced the score and accuracy functions for ℋ_*ij*_ as

(4) $$\begin{array}{c} S\left({ℋ}_{ij}\right)={}^{\prime }{ϒ}_{ij}^{2}-\vartheta_{ij}^{2}, \end{array}$$

where *S*(ℋ_*ij*_) ∈ [−1,1]. It is notified that the score function is unable to differentiate the PFSNs in some cases. For example, let ℋ_11_ = 0.3162,  0.4472 and ℋ_12_ = 0.5477,  0.6324, then according to the definition of score function, we have *S*(ℋ_11_) = −0.1 and *S*(ℋ_12_) = −0.1. So, in this case, it is impossible to find the finest alternative utilizing the score function. To handle this drawback, an accuracy function has been developed in which the sum of squares of membership and nonmembership such as

(5) $$\begin{array}{c} A\left({ℋ}_{ij}\right)={}^{\prime }{ϒ}_{ij}^{2}+\vartheta_{ij}^{2}, \end{array}$$

where *A*(ℋ_*ij*_) ∈ [−1,1].

Thus, to compare two PFSNs ℋ_*ij*_ and ℛ_*ij*_, the following comparison laws are defined:

(1) If *S*(ℋ_*ij*_) > *S*(ℛ_*ij*_), then ℋ_*ij*_ > ℛ_*ij*_
(2) If *S*(ℋ_*ij*_)=*S*(ℛ_*ij*_), then
  - If *A*(ℋ_*ij*_) > *A*(ℛ_*ij*_), then ℋ_*ij*_ > ℛ_*ij*_
  - If *A*(ℋ_*ij*_)=*A*(ℛ_*ij*_), then ℋ_*ij*_=ℛ_*ij*_

Matrices play a vital role in numerous areas of life such as calculation strategies, managing the magnitude of several engineering complications, medical science, and social science.

**Definition 5 (see [39]).**

If (*F*_*A*_, *E*) becomes a soft Pythagorean set softer than *X*, then subset *X* ∈ *E* is defined differently by *R*_*A*_={(*P*, *e*), *e* ∈ *a*, *P* ∈ *F*_*A*_}. Let *R*_*A*_ be identified by its MG and NMG functions such as ′ϒ_*R*_*A*__ : *X* × *E*⟶[0,1] and *ϑ*_*R*_*A*__ : *X* × *E*⟶[0,1].

(6) $$\begin{array}{c} \left[M\right]={\left[{}^{\prime }{ϒ}_{ij}\right]}_{\begin{array}{c} m\\ \alpha \times \beta \end{array}}={\left({}^{\prime }{ϒ}_{ij}^{M},\vartheta_{ij}^{M}\right)}_{\alpha \times \beta }=\left[\begin{array}{ccccc} \left({}^{\prime }{ϒ}_{11},\vartheta_{11}\right) & \left({}^{\prime }{ϒ}_{12},\vartheta_{12}\right) & \ldots & \ldots & \left({}^{\prime }{ϒ}_{1n},\vartheta_{1n}\right)\\ \left({}^{\prime }{ϒ}_{21},\vartheta_{21}\right) & \left({}^{\prime }{ϒ}_{22},\vartheta_{22}\right) & \ldots & \ldots & \left({}^{\prime }{ϒ}_{2n},\vartheta_{2n}\right)\\ \vdots & \vdots & \vdots & \vdots & \vdots \\ \vdots & \vdots & \vdots & \vdots & \vdots \\ \left({}^{\prime }ϒ,\vartheta_{m1}\right) & \left({}^{\prime }{ϒ}_{m2},\vartheta_{m2}\right) & \ldots & \ldots & \left({}^{\prime }{ϒ}_{mn},\vartheta_{mn}\right) \end{array}\right], \end{array}$$

which is called the Pythagorean fuzzy soft matrix (PFSM) of *α* × *β* order.

**Definition 6 (see [39]).**

If *A*=[′ϒ_*ij*_^*A*^ − *ϑ*_*ij*_^*A*^] and *B*=[′ϒ_*ij*_^*B*^ − *ϑ*_*ij*_^*B*^] be two PFSM_*α*×*β*_. Then, score matrix of PFSM of *A* is given as

(7) $$\begin{array}{c} S=\left[s_{ij}\right]=\left[\left({\left({}^{\prime }{ϒ}_{ij}^{A}\right)}^{2}-{\left(\vartheta_{ij}^{A}\right)}^{2}\right)\right],\;\text{for all }i\text{ and }j. \end{array}$$

**Definition 7 (see [39]).**

If *A*=[′ϒ_*ij*_^*A*^ − *ϑ*_*ij*_^*A*^] and *B*=[′ϒ_*ij*_^*B*^ − *ϑ*_*ij*_^*B*^] be two PFSM_*α*×*β*_. Then, the utility matrix for PFSM of *A* and *B* is given as

(8) $$\begin{array}{c} U\left(A,B\right)=\left[S\left(A\right)-S\left(B\right)\right],\;\text{for all }i\text{ and }j. \end{array}$$

## 3. Logical Operators for PFSS with Their Fundamental Properties

In this section, we are going to develop some logical operations such as OR-operation and AND-operation with their desirable properties. Also, we will introduce the necessity and possibility operations with their fundamental characteristics.

**Definition 8 .**

Let (*K*, *A*) = {*P*, (′ϒ_*A*_(*P*), *ϑ*_*A*_(*P*))*|P* ∈ *A*} and (*L*, *B*) = {*P*, (′ϒ_*B*_(*P*), *ϑ*_*B*_(*P*))*|P* ∈ *B*} be two PFSS, where ′ϒ_*A*_(*P*), ′ϒ_*B*_(*P*), *ϑ*_*A*_(*P*), *ϑ*_*B*_(*P*) ∈ [0,1]. Then, OR-operation between them is written as follows:

(9) $$\begin{array}{c} \left(K,A\right)\vee \left(L,B\right)=\left[\left\{P,\max\left({}^{\prime }{ϒ}_{A}\left(P\right),{}^{\prime }{ϒ}_{B}\left(P\right)\right),\min\left(\vartheta_{A}\left(P\right),\vartheta_{B}\left(P\right)\right)|P\in A,B\right\}\right]. \end{array}$$

**Definition 9 .**

Let (*K*, *A*) = {*P*, (′ϒ_*A*_(*P*), *ϑ*_*A*_(*P*))*|P* ∈ *A*} and (*L*, *B*) = {*P*, (′ϒ_*B*_(*P*), *ϑ*_*B*_(*P*))*|P* ∈ *B*} be two PFSS, where ′ϒ_*A*_(*P*), ′ϒ_*B*_(*P*), *ϑ*_*A*_(*P*), *ϑ*_*B*_(*P*) ∈ [0,1]. Then, AND-operation between them is written as follows:

(10) $$\begin{array}{c} \left(K,A\right)\wedge \left(L,B\right)=\left[\left\{P,\min\left({}^{\prime }{ϒ}_{A}\left(P\right),{}^{\prime }{ϒ}_{B}\left(P\right)\right),\max\left(\vartheta_{A}\left(P\right),\vartheta_{B}\left(P\right)\right)|P\in A,B\right\}\right]. \end{array}$$

**Proposition 1 .**

Let (*K*, *A*) = {*P*, (′ϒ_*A*_(*P*), *ϑ*_*A*_(*P*))*|P* ∈ *A*}, (*L*, *B*) = {*P*, (′ϒ_*B*_(*P*), *ϑ*_*B*_(*P*))*|P* ∈ *B*}, and (*M*, *C*) = {*P*, (′ϒ_*C*_(*P*), *ϑ*_*C*_(*P*))*|P* ∈ *C*} be three PFSS ′ϒ_*A*_(*P*), ′ϒ_*B*_(*P*), ′ϒ_*C*_(*P*), *ϑ*_*A*_(*P*), *ϑ*_*B*_(*P*), *ϑ*_*C*_(*P*) ∈ [0,1].

1. (*K*, *A*)∨(*L*, *B*) = (*L*, *B*)∨(*K*, *A*)
2. (*K*, *A*)∨((*L*, *B*)∨(*M*, *C*)) = ((*K*, *A*)∨(*L*, *B*))∨(*M*, *C*)

**Proof**

(1) Let (*K*, *A*) = {*P*, (′ϒ_*A*_(*P*), *ϑ*_*A*_(*P*))*|P* ∈ *A*} and (*L*, *B*) = {*P*, (′ϒ_*B*_(*P*), *ϑ*_*B*_(*P*))*|P* ∈ *B*} be two PFSS, where ′ϒ_*A*_(*P*), ′ϒ_*B*_(*P*), *ϑ*_*A*_(*P*), *ϑ*_*B*_(*P*) ∈ [0,1]. Then, utilizing Definition 8, we get

(11) $$\begin{array}{c} \left(K,A\right)\vee \left(L,B\right)=\left\{P,\left({}^{\prime }{ϒ}_{A}\left(P\right),\vartheta_{A}\left(P\right)\right)|P\in A\right\}\vee \left\{P,\left({}^{\prime }{ϒ}_{B}\left(P\right),\vartheta_{B}\left(P\right)\right)|P\in B\right\},\\ \left(K,A\right)\vee \left(L,B\right)=\left[\left\{P,\max\left({}^{\prime }{ϒ}_{A}\left(P\right),{}^{\prime }{ϒ}_{B}\left(P\right)\right),\min\left(\vartheta_{A}\left(P\right),\vartheta_{B}\left(P\right)\right)|P\in A,B\right\}\right],\\ \left(K,A\right)\vee \left(L,B\right)=\left[\left\{P,\max\left({}^{\prime }{ϒ}_{B}\left(P\right),{}^{\prime }{ϒ}_{A}\left(P\right)\right),\min\left(\vartheta_{B}\left(P\right),\vartheta_{A}\left(P\right)\right)|P\in A,B\right\}\right],\\ \left(K,A\right)\vee \left(L,B\right)=\left\{P,\left({}^{\prime }{ϒ}_{B}\left(P\right),\vartheta_{B}\left(P\right)\right)|P\in B\right\}\vee \left\{P,\left({}^{\prime }{ϒ}_{A}\left(P\right),\vartheta_{A}\left(P\right)\right)|P\in A\right\},\\ \left(K,A\right)\vee \left(L,B\right)=\left(L,B\right)\vee \left(K,A\right). \end{array}$$

**Proof**

(2) Let (*K*, *A*) = {*P*, (′ϒ_*A*_(*P*), *ϑ*_*A*_(*P*))*|P* ∈ *A*}, (*L*, *B*) =  {*P*, (′ϒ_*B*_(*P*), *ϑ*_*B*_(*P*))*|P* ∈ *B*}, and (*M*, *C*) =  {*P*, (′ϒ_*C*_(*P*), *ϑ*_*C*_(*P*))*|P* ∈ *C*} be three PFSS ′ϒ_*A*_(*P*), ′ϒ_*B*_(*P*), *ϑ*_*A*_(*P*), *ϑ*_*B*_(*P*) ∈ [0,1].

(12) $$\begin{array}{c} \left(K,A\right)\vee \left(\left(L,B\right)\vee \left(M,C\right)\right)=\left\{P,\;\left({}^{\prime }{ϒ}_{A}\left(P\right),\vartheta_{A}\left(P\right)\right)|P\in A\right\}\vee \left(\left\{P,\left({}^{\prime }{ϒ}_{B}\left(P\right),\vartheta_{B}\left(P\right)\right)|P\in B\right\}\vee \left\{P,\left({}^{\prime }{ϒ}_{C}\left(P\right),\vartheta_{C}\left(P\right)\right)|P\in C\right\}\right)\\ =\left\{P,\left({}^{\prime }{ϒ}_{A}\left(P\right),\vartheta_{A}\left(P\right)\right)|P\in A\right\}\vee \left(\left\{P,\max\left({}^{\prime }{ϒ}_{B}\left(P\right),{}^{\prime }{ϒ}_{C}\left(P\right)\right),\min\left(\vartheta_{B}\left(P\right),\vartheta_{C}\left(P\right)\right)|P\in B,C\right\}\right)\\ =\left(\left\{P,\max\left({}^{\prime }{ϒ}_{A}\left(P\right),\max\left({}^{\prime }{ϒ}_{B}\left(P\right),{}^{\prime }ϒ\left(P\right)\right)\right),\min\left(\vartheta_{A}\left(P\right),\min\left(\vartheta_{B}\left(P\right)|,|\vartheta_{C}|\left(P\right)\right)\right)|P\in A,B,C\right\}\right)\\ =\left(\left\{P,\max\left({}^{\prime }{ϒ}_{A}\left(P\right),\left({}^{\prime }{ϒ}_{B}\left(P\right),{}^{\prime }{ϒ}_{C}\left(P\right)\right)\right),\min\left(\vartheta_{A}\left(P\right),\left(\vartheta_{B}\left(P\right)|,|\vartheta_{C}|\left(P\right)\right)\right)|P\in A,B,C\right\}\right)\\ =\left(\left\{P,\max\left({}^{\prime }{ϒ}_{A}\left(P\right),{}^{\prime }{ϒ}_{B}\left(P\right),{}^{\prime }{ϒ}_{C}\left(P\right)\right),\min\left(\vartheta_{A}\left(P\right),\vartheta_{B}\left(P\right),\vartheta_{C}\left(P\right)\right)|P\in A,B,C\right\}\right)\\ =\left(\left(K,A\right)\vee \left(L,B\right)\right)\vee \left(M,C\right)\\ =\left(\left\{P,\left({}^{\prime }{ϒ}_{A}\left(P\right),\vartheta_{A}\left(P\right)\right)|P\in A\right\}\vee \left\{P,\left({}^{\prime }{ϒ}_{B}\left(P\right),\vartheta_{B}\left(P\right)\right)|P\in B\right\}\right)\vee \left\{P,\left({}^{\prime }{ϒ}_{C}\left(P\right),\vartheta_{C}\left(P\right)\right)|P\in C\right\}\\ =\left(\left\{P,\max\left({}^{\prime }{ϒ}_{A}\left(P\right),{}^{\prime }{ϒ}_{B}\left(P\right)\right),\min\left(\vartheta_{A}\left(P\right),\vartheta_{B}\left(P\right)\right)|P\in A,B\right\}\right)\vee \left\{P,\left({}^{\prime }{ϒ}_{C}\left(P\right),\vartheta_{C}\left(P\right)\right)|P\in C\right\}\\ =\left(\left\{P,\max\left(\max\left({}^{\prime }{ϒ}_{A}\left(P\right),{}^{\prime }{ϒ}_{B}\left(P\right)\right),{}^{\prime }{ϒ}_{C}\left(P\right)\right),\min\left(\min\left(\vartheta_{A}\left(P\right),\vartheta_{B}\left(P\right)\right),\vartheta_{C}\left(P\right)\right)|P\in A,B,C\right\}\right)\\ =\left(\left\{P,\max\left({}^{\prime }{ϒ}_{A}\left(P\right),{}^{\prime }{ϒ}_{B}\left(P\right),{}^{\prime }{ϒ}_{C}\left(P\right)\right),\min\left(\vartheta_{A}\left(P\right),\vartheta_{B}\left(P\right),\vartheta_{C}\left(P\right)\right)|P\in A,B,C\right\}\right). \end{array}$$

Hence,

(13) $$\begin{array}{c} \left(K,A\right)\vee \left(\left(L,B\right)\vee \left(M,C\right)\right)=\left(\left(K,A\right)\vee \left(L,B\right)\right)\vee \left(M,C\right). \end{array}$$

**Proposition 2 .**

Let (*K*, *A*) = {*P*, (′ϒ_*A*_(*P*), *ϑ*_*A*_(*P*))*|P* ∈ *A*}, (*L*, *B*) = {*P*, (′ϒ_*B*_(*P*), *ϑ*_*B*_(*P*))*|P* ∈ *B*}, and (*M*, *C*) = {*P*, (′ϒ_*C*_(*P*), *ϑ*_*C*_(*P*))*|P* ∈ *A*} be three PFSS *μ*_*A*_(*P*), *μ*_*B*_(*P*), *ϑ*_*A*_(*P*), *ϑ*_*B*_(*P*) ∈ [0,1].

1. (*K*, *A*)∧(*L*, *B*) = (*L*, *B*)∧(*K*, *A*)
2. (*K*, *A*)∧((*L*, *B*)∧(*M*, *C*)) = ((*K*, *A*)∧(*L*, *B*))∧(*M*, *C*)

**Proof**

(1) Let (*K*, *A*) = {*P*, (′ϒ_*A*_(*P*), *ϑ*_*A*_(*P*))*|P* ∈ *A*} and (*L*, *B*) = {*P*, (′ϒ_*B*_(*P*), *ϑ*_*B*_(*P*))*|P* ∈ *B*} be two PFSS, where ′ϒ_*A*_(*P*), ′ϒ_*B*_(*P*), *ϑ*_*A*_(*P*), *ϑ*_*B*_(*P*) ∈ [0,1]. Then, using Definition 9, we get

(14) $$\begin{array}{c} \left(K,A\right)\wedge \left(L,B\right)=\left\{P,\left({}^{\prime }{ϒ}_{A}\left(P\right),\vartheta_{A}\left(P\right)\right)|P\in A\right\}\wedge \left\{P,\left({}^{\prime }{ϒ}_{B}\left(P\right),\vartheta_{B}\left(P\right)\right)|P\in B\right\},\\ \left(K,A\right)\wedge \left(L,B\right)=\left[\left\{P,\min\left({}^{\prime }{ϒ}_{A}\left(P\right),{}^{\prime }{ϒ}_{B}\left(P\right)\right),\max\left(\vartheta_{A}\left(P\right),\vartheta_{B}\left(P\right)\right)|P\in A,B\right\}\right],\\ \left(K,A\right)\wedge \left(L,B\right)=\left[\left\{P,\min\left({}^{\prime }{ϒ}_{B}\left(P\right),{}^{\prime }{ϒ}_{A}\left(P\right)\right),\max\left(\vartheta_{B}\left(P\right),\vartheta_{A}\left(P\right)\right)|P\in A,B\right\}\right],\\ \left(K,A\right)\wedge \left(L,B\right)=\left\{P,\left({}^{\prime }{ϒ}_{B}\left(P\right),\vartheta_{B}\left(P\right)\right)|P\in B\right\}\wedge \left\{P,\left({}^{\prime }{ϒ}_{A}\left(P\right),\vartheta_{A}\left(P\right)\right)|P\in A\right\},\\ \left(K,A\right)\wedge \left(L,B\right)=\left(L,B\right)\wedge \left(K,A\right). \end{array}$$

**Proof**

(2) Let (*K*, *A*) = {*P*, (′ϒ_*A*_(*P*), *ϑ*_*A*_(*P*))*|P* ∈ *A*}, (*L*, *B*) = {*P*, (′ϒ_*B*_(*P*), *ϑ*_*B*_(*P*))*|P* ∈ *B*}, and (*M*, *C*) = {*P*, (′ϒ_*C*_(*P*), *ϑ*_*C*_(*P*))*|P* ∈ *C*} be three PFSS ′ϒ_*A*_(*P*), ′ϒ_*B*_(*P*), ′ϒ_*C*_(*P*), *ϑ*_*A*_(*P*), *ϑ*_*B*_(*P*), *ϑ*_*c*_(*P*) ∈ [0,1]. Then, same as the above utilizing Definition 9, we can get the required result.

**Proposition 3 .**

Let (*K*, *A*) = {*P*, (′ϒ_*A*_(*P*), *ϑ*_*A*_(*P*))*|P* ∈ *A*} and (*L*, *B*) = {*P*, (′ϒ_*B*_(*P*), *ϑ*_*B*_(*P*))*|P* ∈ *B*} be two PFSS. Then, De Morgan Laws are given as follows:

1. ((*K*, *A*)∨(*L*, *B*))^0^=(*K*, *A*)^*O*^∧(*L*, *B*)^*O*^
2. ((*K*, *A*)∧(*L*, *B*))^0^=(*K*, *A*)^*O*^∨(*L*, *B*)^*O*^

**Proof**

(1) Let (*K*, *A*) = {*P*, (′ϒ_*A*_(*P*), *ϑ*_*A*_(*P*))*|P* ∈ *A*} and (*L*, *B*) = {*P*, (′ϒ_*B*_(*P*), *ϑ*_*B*_(*P*))*|P* ∈ *B*} be two PFSS. Then,

(15) $$\begin{array}{c} {\left(\left(K,A\right)\vee \left(L,B\right)\right)}^{c}={\left(\left\{P,\left({}^{\prime }{ϒ}_{A}\left(P\right),\vartheta_{A}\left(P\right)\right)|P\in A\right\}\vee \left\{P,\left({}^{\prime }{ϒ}_{B}\left(P\right),\vartheta_{B}\left(P\right)\right)|P\in B\right\}\right)}^{O}\\ ={\left(\left\{P,\max\left({}^{\prime }{ϒ}_{A}\left(P\right),{}^{\prime }{ϒ}_{B}\left(P\right)\right),\min\left(\vartheta_{A}\left(P\right),\vartheta_{B}\left(P\right)\right)|P\in A,B\right\}\right)}^{O}\\ =\left(\left\{P,\min\left(\vartheta_{A}\left(P\right),\vartheta_{B}\left(P\right)\right),\max\left({}^{\prime }{ϒ}_{A}\left(P\right),{}^{\prime }{ϒ}_{B}\left(P\right)\right)|P\in A,B\right\}\right),\\ {\left(K,A\right)}^{O}\wedge {\left(L,B\right)}^{O}={\left\{P,\left({}^{\prime }{ϒ}_{A}\left(P\right),\vartheta_{A}\left(P\right)\right)|P\in A\right\}}^{o}\wedge {\left\{P,\left({}^{\prime }{ϒ}_{B}\left(P\right),\vartheta_{B}\left(P\right)\right)|P\in B\right\}}^{o}\\ =\left\{P,\left(\vartheta_{A}\left(P\right),{}^{\prime }{ϒ}_{A}\left(P\right)\right)|P\in A\right\}\wedge \left\{P,\left(\vartheta_{B}\left(P\right),{}^{\prime }{ϒ}_{B}\left(P\right)\right)|P\in B\right\}\\ =\left(\left\{P,\min\left(\vartheta_{A}\left(P\right),\vartheta_{B}\left(P\right)\right),\max\left({}^{\prime }{ϒ}_{A}\left(P\right),{}^{\prime }{ϒ}_{B}\left(P\right)\right)|P\in A,B\right\}\right). \end{array}$$

Hence proved.

(16) $$\begin{array}{c} {\left(\left(K,A\right)\vee \left(L,B\right)\right)}^{0}={\left(K,A\right)}^{O}\wedge {\left(L,B\right)}^{O}. \end{array}$$

**Proof**

(2) Let (*K*, *A*) = {*P*, (′ϒ_*A*_(*P*), *ϑ*_*A*_(*P*))*|P* ∈ *A*} and (*L*, *B*) = {*P*, (′ϒ_*B*_(*P*), *ϑ*_*B*_(*P*))*|P* ∈ *B*} be two PFSS. Then,

(17) $$\begin{array}{c} {\left(\left(K,A\right)\wedge \left(L,B\right)\right)}^{0}={\left(\left\{P,\left({}^{\prime }{ϒ}_{A}\left(P\right),\vartheta_{A}\left(P\right)\right)|P\in A\right\}\wedge \left\{P,\left({}^{\prime }{ϒ}_{B}\left(P\right),\vartheta_{B}\left(P\right)\right)|P\in B\right\}\right)}^{O}\\ ={\left(\left\{P,\min\left({}^{\prime }{ϒ}_{A}\left(P\right),{}^{\prime }{ϒ}_{B}\left(P\right)\right),\max\left(\vartheta_{A}\left(P\right),\vartheta_{B}\left(P\right)\right)|P\in A,B\right\}\right)}^{O}\\ =\left(\left\{P,\max\left(\vartheta_{A}\left(P\right),\vartheta_{B}\left(P\right)\right),\min\left({}^{\prime }{ϒ}_{A}\left(P\right),{}^{\prime }{ϒ}_{B}\left(P\right)\right)|P\in A,B\right\}\right),\\ {\left(K,A\right)}^{O}\vee {\left(L,B\right)}^{O}={\left\{P,\left({}^{\prime }{ϒ}_{A}\left(P\right),\vartheta_{A}\left(P\right)\right)|P\in A\right\}}^{o}\vee {\left\{P,\left({}^{\prime }{ϒ}_{B}\left(P\right),\vartheta_{B}\left(P\right)\right)|P\in B\right\}}^{o}\\ =\left\{P,\left(\vartheta_{A}\left(P\right),{}^{\prime }{ϒ}_{A}\left(P\right)\right)|P\in A\right\}\vee \left\{P,\left(\vartheta_{B}\left(P\right),{}^{\prime }{ϒ}_{B}\left(P\right)\right)|P\in B\right\}\\ =\left(\left\{P,\max\left(\vartheta_{A}\left(P\right),\vartheta_{B}\left(P\right)\right),\min\left({}^{\prime }{ϒ}_{A}\left(P\right),{}^{\prime }{ϒ}_{B}\left(P\right)\right)|P\in A,B\right\}\right). \end{array}$$

Hence proved.

(18) $$\begin{array}{c} {\left(\left(K,A\right)\wedge \left(L,B\right)\right)}^{0}={\left(K,A\right)}^{O}\vee {\left(L,B\right)}^{O}. \end{array}$$

**Definition 10 .**

Let (*K*, *A*) = {*P*, (′ϒ_*A*_(*P*), *ϑ*_*A*_(*P*))*|P* ∈ *A*} be a PFSS. Then, the necessity operation on PFSS is denoted by   ⊕  (*K*, *A*) and defined as follows:

(19) $$\begin{array}{c} \;⊕\;\left(K,A\right)=\left\{{}^{\prime }{ϒ}_{A}\left(P\right),\sqrt{\left(1-{}^{\prime }{ϒ}_{A}{\left(P\right)}^{2}\right)}\right\}. \end{array}$$

**Definition 11 .**

Let (*K*, *A*) = {*P*, (′ϒ_*A*_(*P*), *ϑ*_*A*_(*P*))*|P* ∈ *A*} be a PFSS, then the possibility operation on PFSS is denoted by ⊗(*K*, *A*) and written as follows:

(20) $$\begin{array}{c} ⊗\left(K,A\right)=\left\{\sqrt{\left(1-\vartheta_{A}{\left(P\right)}^{2}\right)},\vartheta_{A}\left(P\right)\right\}. \end{array}$$

**Proposition 4 .**

Let (*K*, *A*) = {*P*, (′ϒ_*A*_(*P*), *ϑ*_*A*_(*P*))*|P* ∈ *A*} be a PFSS. Then, the following properties are satisfied.

1. [  ⊕  (*K*, *A*)^*O*^]^*O*^ = ⊗(*K*, *A*)
2. [⊗(*K*, *A*)^*O*^]^*O*^ = ⊕  (*K*, *A*)
3.   ⊕  [  ⊕  (*K*, *A*)] = ⊕  (*K*, *A*)
4.   ⊕  [⊗(*K*, *A*)] = ⊗(*K*, *A*)
5. ⊗[  ⊕  (*K*, *A*)] = ⊕  (*K*, *A*)
6. ⊗[⊗(*K*, *A*)] = ⊗(*K*, *A*)

**Proof**

(1) Let (*K*, *A*) = {*P*, (′ϒ_*A*_(*P*), *ϑ*_*A*_(*P*))*|P* ∈ *A*} be a PFSS. Then,

(21) $$\begin{array}{c} {\left[\;⊕\;{\left(K,\;A\right)}^{O}\right]}^{O}={\left[\;⊕\;{\left\{P,\left({}^{\prime }{ϒ}_{A}\left(P\right),\vartheta_{A}\left(P\right)\right)|P\in A\right\}}^{o}\right]}^{O}\\ ={\left[\;⊕\;\left\{\left\{P,\left(\vartheta_{A}\left(P\right),{}^{\prime }{ϒ}_{A}\left(P\right)\right)|P\in A\right\}\right\}\right]}^{o}\\ ={\left\{P,\left(\vartheta_{A}\left(P\right),\sqrt{\left(1-{\left(\vartheta_{A}\left(P\right)\right)}^{2}\right)}\right)|P\in A\right\}}^{o}\\ =\left\{P,\left(\sqrt{\left(1-{\left(\vartheta_{A}\left(P\right)\right)}^{2}\right)},\vartheta_{A}\left(P\right)\right)|P\in A\right\}\\ =⊗\left(K,A\right). \end{array}$$

**Proof**

(2) Let (*K*, *A*) = {*P*, (′ϒ_*A*_(*P*), *ϑ*_*A*_(*P*))*|P* ∈ *A*} be a PFSS. Then,

(22) $$\begin{array}{c} {\left[⊗{\left(K,\;A\right)}^{O}\right]}^{O}={\left[⊗{\left\{P,\left({}^{\prime }{ϒ}_{A}\left(P\right),\vartheta_{A}\left(P\right)\right)|P\in A\right\}}^{o}\right]}^{O}\\ ={\left[⊗\left\{\left\{P,\left(\vartheta_{A}\left(P\right),{}^{\prime }{ϒ}_{A}\left(P\right)\right)|P\in A\right\}\right\}\right]}^{o}\\ ={\left\{P,\left(\sqrt{\left(1-{}^{\prime }{ϒ}_{A}{\left(P\right)}^{2}\right)},{}^{\prime }{ϒ}_{A}\left(P\right)\right)|P\in A\right\}}^{o}\\ =\left\{P,\left({}^{\prime }{ϒ}_{A}\left(P\right),\sqrt{\left(1-{}^{\prime }{ϒ}_{A}{\left(P\right)}^{2}\right)}\right)|P\in A\right\}\\ =\;⊕\;\left(K,A\right). \end{array}$$

**Proof**

(3) Let (*K*, *A*) = {*P*, (′ϒ_*A*_(*P*), *ϑ*_*A*_(*P*))*|P* ∈ *A*} be a PFSS. Then,

(23) $$\begin{array}{c} \;⊕\;\left[\;⊕\;\left(K,A\right)\right]=\;⊕\;\left[\;⊕\;\left\{P,\left({}^{\prime }{ϒ}_{A}\left(P\right),\vartheta_{A}\left(P\right)\right)|P\in A\right\}\right]\\ =\;⊕\;\left\{P,\left({}^{\prime }{ϒ}_{A}\left(P\right),\sqrt{\left(1-{}^{\prime }{ϒ}_{A}{\left(P\right)}^{2}\right)}\right)|P\in A\right\}\\ =\left\{P,\left({}^{\prime }{ϒ}_{A}\left(P\right),\sqrt{\left(1-{}^{\prime }{ϒ}_{A}{\left(P\right)}^{2}\right)}\right)|P\in A\right\}\\ =\;⊕\;\left(K,A\right). \end{array}$$

**Proof**

(4) Let (*K*, *A*) = {*P*, (′ϒ_*A*_(*P*), *ϑ*_*A*_(*P*))*|P* ∈ *A*} be a PFSS. Then,

(24) $$\begin{array}{c} \;⊕\;\left[⊗\left(K,A\right)\right]=⊗\left[⊗\left\{P,\left({}^{\prime }{ϒ}_{A}\left(P\right),\vartheta_{A}\left(P\right)\right)|P\in A\right\}\right]\\ =⊗\left\{P,\sqrt{\left(1-\vartheta_{A}{\left(P\right)}^{2}\right)},\vartheta_{A}\left(P\right)|P\in A\right\}\\ =\left[P,\left(\sqrt{\left(1-\vartheta_{A}{\left(P\right)}^{2}\right)},\sqrt{1-{\left(\sqrt{\left(1-\vartheta_{A}{\left(P\right)}^{2}\right)}\right)}^{2}}\right)|P\in A\right]\\ =\left\{P,\left(\sqrt{\left(1-\vartheta_{A}{\left(P\right)}^{2}\right)},\sqrt{1-\left(1-\vartheta_{A}{\left(P\right)}^{2}\right)}\right)|P\in A\right\}\\ =\left\{P,\sqrt{\left(1-\vartheta_{A}{\left(P\right)}^{2}\right)},\vartheta_{A}\left(P\right)|P\in A\right\}\\ =⊗\left(K,A\right). \end{array}$$

**Proof**

(5) Let (*K*, *A*) = {*P*, (′ϒ_*A*_(*P*), *ϑ*_*A*_(*P*))*|P* ∈ *A*} be a PFSS. Then,

(25) $$\begin{array}{c} ⊗\left[\;⊕\;\left(K,A\right)\right]=⊗\left[\;⊕\;\left\{P,\left({}^{\prime }{ϒ}_{A}\left(P\right),\vartheta_{A}\left(P\right)\right)|P\in A\right\}\right]\\ =⊗\left\{P,\left({}^{\prime }{ϒ}_{A}\left(P\right),\sqrt{\left(1-{}^{\prime }{ϒ}_{A}{\left(P\right)}^{2}\right)}\right)|P\in A\right\}\\ =\left[P,\left(\sqrt{1-{\left(\sqrt{1-{}^{\prime }{ϒ}_{A}{\left(P\right)}^{2}}\right)}^{2}},\sqrt{\left(1-{}^{\prime }{ϒ}_{A}{\left(P\right)}^{2}\right)}\right)|P\in A\right]\\ =\left\{P,\left({}^{\prime }{ϒ}_{A}\left(P\right),\sqrt{\left(1-{}^{\prime }{ϒ}_{A}{\left(P\right)}^{2}\right)}\right)|P\in A\right\}\\ =\;⊕\;\left(K,A\right). \end{array}$$

**Proof**

(6) Let (*K*, *A*) = {*P*, (′ϒ_*A*_(*P*), *ϑ*_*A*_(*P*))*|P* ∈ *A*} be a PFSS. Then,

(26) $$\begin{array}{c} ⊗\left[⊗\left(K,A\right)\right]=⊗\left[⊗\left\{P,\left({}^{\prime }{ϒ}_{A}\left(P\right),\vartheta_{A}\left(P\right)\right)|P\in A\right\}\right]\\ =⊗\left\{P,\left(\sqrt{\left(1-\vartheta_{A}{\left(P\right)}^{2}\right)},\vartheta_{A}\left(P\right)\right)|P\in A\right\}\\ =\left\{P,\left(\sqrt{\left(1-\vartheta_{A}{\left(P\right)}^{2}\right)},\vartheta_{A}\left(P\right)\right)|P\in A\right\}\\ =⊗\left(K,A\right). \end{array}$$

**Proposition 5 .**

Let (*K*, *A*) = {*P*, (′ϒ_*A*_(*P*), *ϑ*_*A*_(*P*))*|P* ∈ *A*} and (*L*, *B*) =  {*P*, (′ϒ_*B*_(*P*), *ϑ*_*B*_(*P*))*|P* ∈ *B*} be two PFSS. Then,

1.   ⊕  [(*K*, *A*)∧(*L*, *B*)] = ⊕  (*K*, *A*)∧  ⊕  (*L*, *B*)
2.   ⊕  [(*K*, *A*)∨(*L*, *B*)]=  ⊕  (*K*, *A*)∨  ⊕  (*L*, *B*)

**Proof**

(1) Let (*K*, *A*) = {*P*, (′ϒ_*A*_(*P*), *ϑ*_*A*_(*P*))*|P* ∈ *A*} and (*L*, *B*) = {*P*, (′ϒ_*B*_(*P*), *ϑ*_*B*_(*P*))*|P* ∈ *B*} be two PFSS.

(27) $$\begin{array}{c} \;⊕\;\left[\left(K,A\right)\wedge \left(L,B\right)\right]=\;⊕\;\left[P,\min\left({}^{\prime }{ϒ}_{A}\left(P\right),{}^{\prime }{ϒ}_{B}\left(P\right)\right),\max\left(\vartheta_{A}\left(P\right),\vartheta_{B}\left(P\right)\right)|P\in A,B\right]\\ =\left[P,\left\{\min\left({}^{\prime }{ϒ}_{A}\left(P\right),{}^{\prime }{ϒ}_{B}\left(P\right)\right),\sqrt{1-\min\left({}^{\prime }{ϒ}_{A}{\left(P\right)}^{2},{}^{\prime }{ϒ}_{B}{\left(P\right)}^{2}\right)}\right\}|P\in A,B\right]\\ =\left[P,\left\{\min\left({}^{\prime }{ϒ}_{A}\left(P\right),{}^{\prime }{ϒ}_{B}\left(P\right)\right),\max\sqrt{\left(1-{}^{\prime }{ϒ}_{A}{\left(P\right)}^{2},1-{}^{\prime }{ϒ}_{B}{\left(P\right)}^{2}\right)|}\right\}|P\in A,B\right]\\ =\left\{P,\left({}^{\prime }{ϒ}_{A}\left(P\right),\sqrt{\left(1-{}^{\prime }{ϒ}_{A}{\left(P\right)}^{2}\right)}\right)|P\in A\right\}\wedge \left\{P,\left({}^{\prime }{ϒ}_{B}\left(P\right),\sqrt{\left(1-{}^{\prime }{ϒ}_{B}{\left(P\right)}^{2}\right)}\right)|P\in A,B\right\}\\ =\;⊕\;\left(K,A\right)\wedge \;⊕\;\left(L,B\right). \end{array}$$

**Proof**

(2) Let (*K*, *A*) = {*P*, (′ϒ_*A*_(*P*), *ϑ*_*A*_(*P*))*|P* ∈ *A*} and (*L*, *B*) = {*P*, (′ϒ_*B*_(*P*), *ϑ*_*B*_(*P*))*|P* ∈ *B*} be two PFSS.

(28) $$\begin{array}{c} \;⊕\;\left[\left(K,A\right)\vee \left(L,B\right)\right]=\;⊕\;\left[P,\max\left({}^{\prime }{ϒ}_{A}\left(P\right),{}^{\prime }{ϒ}_{B}\left(P\right)\right),\min\left(\vartheta_{A}\left(P\right),\vartheta_{B}\left(P\right)\right)|P\in A,B\right]\\ =\left[P,\left\{\max\left({}^{\prime }{ϒ}_{A}\left(P\right),{}^{\prime }{ϒ}_{B}\left(P\right)\right),\sqrt{1-\max\left({}^{\prime }{ϒ}_{A}{\left(P\right)}^{2},{}^{\prime }{ϒ}_{B}{\left(P\right)}^{2}\right)}\right\}|P\in A,B\right]\\ =\left[P,\left\{\max\left({}^{\prime }{ϒ}_{A}\left(P\right),{}^{\prime }{ϒ}_{B}\left(P\right)\right),\min\sqrt{\left(1-{}^{\prime }{ϒ}_{A}{\left(P\right)}^{2},1-{}^{\prime }{ϒ}_{B}{\left(P\right)}^{2}\right)|}\right\}|P\in A,B\right]\\ =\left\{P,\left({}^{\prime }{ϒ}_{A}\left(P\right),\sqrt{\left(1-{}^{\prime }{ϒ}_{A}{\left(P\right)}^{2}\right)}\right)|P\in A\right\}\vee \left\{P,\left({}^{\prime }{ϒ}_{B}\left(P\right),\sqrt{\left(1-{}^{\prime }{ϒ}_{B}{\left(P\right)}^{2}\right)}\right)|P\in A,B\right\}\\ =\;⊕\;\left(K,A\right)\vee \;⊕\;\left(L,B\right). \end{array}$$

**Proposition 6 .**

Let (*K*, *A*) = {*P*, (′ϒ_*A*_(*P*), *ϑ*_*A*_(*P*))*|P* ∈ *A*} and (*L*, *B*) = {*P*, (′ϒ_*B*_(*P*), *ϑ*_*B*_(*P*))*|P* ∈ *B*} be two PFSS. Then,

1. ⊗[(*K*, *A*)∧(*L*, *B*)]=⊗(*K*, *A*)∧⊗(*L*, *B*)
2. ⊗[(*K*, *A*)∨(*L*, *B*)]=⊗(*K*, *A*)∨⊗(*L*, *B*)

**Proof**

(1) Let (*K*, *A*)={*P*, (′ϒ_*A*_(*P*), *ϑ*_*A*_(*P*))*|P* ∈ *A*} and (*L*, *B*)={*P*, (′ϒ_*B*_(*P*), *ϑ*_*B*_(*P*))*|P* ∈ *B*} be two PFSS.

(29) $$\begin{array}{c} ⊗\left[\left(K,A\right)\wedge \left(L,B\right)\right]=\left[P,\min\left({}^{\prime }{ϒ}_{A}\left(P\right),{}^{\prime }{ϒ}_{B}\left(P\right)\right),\max\left(\vartheta_{A}\left(P\right),\vartheta_{B}\left(P\right)\right)|P\in A,B\right]\\ =\left[P,\left\{\sqrt{1-\max\left(\vartheta_{A}{\left(P\right)}^{2},\vartheta_{B}{\left(P\right)}^{2}\right)},\max\left(\vartheta_{A}\left(P\right),\vartheta_{B}\left(P\right)\right)\right\}|P\in A,B\right]\\ =\left[P,\left\{\min\sqrt{\left(1-\vartheta_{A}{\left(P\right)}^{2},1-\vartheta_{B}{\left(P\right)}^{2}\right)},\max\left(\vartheta_{A}\left(P\right),\vartheta_{B}\left(P\right)\right)\right\}|P\in A,B\right]\\ =\left\{P,\left(\sqrt{\left(1-\vartheta_{A}{\left(P\right)}^{2}\right)},\vartheta_{A}\left(P\right)\right)|P\in A\right\}\wedge \left\{P,\left(\sqrt{1-\vartheta_{B}\left(P\right)},\vartheta_{B}\left(P\right)\right)|P\in A,B\right\}\\ =⊗A\wedge ⊗B. \end{array}$$

**Proof**

(2) Similar to assertion 1.

## 4. A Decision-Making Approach for Pythagorean Fuzzy Soft Set Using Score Matrix

In this section, a decision-making approach has been developed to solve decision-making complications based on the score matrix of PFSS. A numerical illustration has been presented to ensure the practicality of our proposed approach.

### 4.1. Proposed Approach

A group of decision-makers intends to select a suitable alternative against *α* variety of substitutes. They choose *β* attributes to select a more suitable alternative. If someone has an additional subdivision of an attribute that forms a relationship like PFSM, each decision-maker grants his inclination individually, substitution as stated in subdivisions of the attributes considered in the PFSM form, and order *α* × *β* gets by PFSM. With this PFSM, we compute matrices values that help to derive the score matrix, along with completely we compute total score regarding every last one alternative by using score matrix. Score functions remain matrix which corresponds to the entire characteristic belonging to a real matrix. The utility matrix is also a real matrix derived from the score function. Finally, using the rank of the alternatives, build the total score matrix. The above process can also be presented is as follows.

### 4.2. Algorithm for the Proposed Approach

  Step 1: input PFSS and create a PFSM
  Step 2: compute the score matrix *S*(*A*), *S*(*B*), *S*(*C*),  and *S*(*D*) by using Definition 6
  Step 3: find a utility matrix by using Definition 7
  Step 4: enumerate the total score matrix
  Step 5: selection belonging to the most suitable alternative
  Step 6: ranking of the alternative builds its total score matrix

### 4.3. Numerical Example

Let *A* and *B* be two PFSMs obtained from PFSS (*F*_*A*_, *E*) and (*G*_*B*_, *E*), respectively. Let *U*={(honda)*g*_1_, (pak hero)*g*_2_, (united)*g*_3_, (sohrab)*g*_4_, (metro)*g*_5_, (king hero)*g*_6_} be a set of bikes of different brands and *E*={*q*_1_, *q*_2_, *q*_3_, *q*_4_, *q*_5_, *q*_6_} be the set of attributes, where *q*_1_=price *q*_2_=environment friendly, *q*_3_=mileage, *q*_4_=engine quality, *q*_5_=structure quality, and *q*_6_=resale value. An investor constituted a committee of decision-makers [*A*, *B*, *C*, *D*] to choose the best bike available in the market. Then, PFSM can be written by using parameters as follows:

(30) $$\begin{array}{c} \left(F_{A},E\right)=\left\{\begin{array}{c} \left\{F\left(q_{1}\right)=\left\{\left(g_{1},0.5,0.7\right),\left(g_{2},0.8,0.5\right),\left(g_{3},0.7,0.4\right),\left(g_{4},0.7,0.6\right),\left(g_{5},0.6,0.6\right),\left(g_{6},0.5,0.7\right)\right\}\right\}\\ \left\{F\left(q_{2}\right)=\left\{\left(g_{1},0.8,0.2\right),\left(g_{2},0.5,0.5\right),\left(g_{3},0.6,0.7\right),\left(g_{4},0.5,0.6\right),\left(g_{5},0.5,0.5\right),\left(g_{6},0.5,0.8\right)\right\}\right\}\\ \left\{F\left(q_{3}\right)=\left\{\left(g_{1},0.8,0.3\right),\left(g_{2},0.4,0.5\right),\left(g_{3},0.6,0.4\right),\left(g_{4},0.5,0.5\right),\left(g_{5},0.5,0.6\right),\left(g_{6},0.4,0.7\right)\right\}\right\}\\ \left\{F\left(q_{4}\right)=\left\{\left(g_{1},0.9,0.1\right),\left(g_{2},0.4,0.7\right),\left(g_{3},0.6,0.7\right),\left(g_{4},0.7,0.6\right),\left(g_{5},0.5,0.5\right),\left(g_{6},0.5,0.6\right)\right\}\right\}\\ \left\{F\left(q_{5}\right)=\left\{\left(g_{1},0.9,0.3\right),\left(g_{2},0.6,0.5\right),\left(g_{3},0.7,0.4\right),\left(g_{4},0.5,0.4\right),\left(g_{5},0.5,0.4\right),\left(g_{6},0.6,0.3\right)\right\}\right\}\\ \left\{F\left(q_{6}\right)=\left\{\left(g_{1},0.8,0.4\right),\left(g_{2},0.4,0.7\right),\left(g_{3},0.6,0.4\right),\left(g_{4},0.4,0.5\right),\left(g_{5},0.5,0.6\right),\left(g_{6},0.3,0.6\right)\right\}\right\} \end{array}\right\},\\ \left(G_{B},E\right)=\left\{\begin{array}{c} \left\{G\left(q_{1}\right)=\left\{\left(g_{1},0.4,0.8\right),\left(g_{2},0.8,0.6\right),\left(g_{3},0.7,0.3\right),\left(g_{4},0.6,0.6\right),\left(g_{5},0.6,0.6\right),\left(g_{6},0.7,0.7\right)\right\}\right\}\\ \left\{G\left(q_{2}\right)=\left\{\left(g_{1},0.7,0.2\right),\left(g_{2},0.5,0.5\right),\left(g_{3},0.6,0.7\right),\left(g_{4},0.5,0.3\right),\left(g_{5},0.4,0.7\right),\left(g_{6},0.4,0.8\right)\right\}\right\}\\ \left\{G\left(q_{3}\right)=\left\{\left(g_{1},0.8,0.3\right),\left(g_{2},0.4,0.7\right),\left(g_{3},0.5,0.4\right),\left(g_{4},0.5,0.5\right),\left(g_{5},0.4,0.6\right),\left(g_{6},0.5,0.7\right)\right\}\right\}\\ \left\{G\left(q_{4}\right)=\left\{\left(g_{1},0.8,0.3\right),\left(g_{2},0.6,0.7\right),\left(g_{3},0.7,0.7\right),\left(g_{4},0.5,0.6\right),\left(g_{5},0.5,0.7\right),\left(g_{6},0.5,0.4\right)\right\}\right\}\\ \left\{G\left(q_{5}\right)=\left\{\left(g_{1},0.7,0.3\right),\left(g_{2},0.6,0.6\right),\left(g_{3},0.5,0.4\right),\left(g_{4},0.5,0.4\right),\left(g_{5},0.6,0.4\right),\left(g_{6},0.5,0.3\right)\right\}\right\}\\ \left\{G\left(q_{6}\right)=\left\{\left(g_{1},0.8,0.6\right),\left(g_{2},0.5,0.7\right),\left(g_{3},0.6,0.4\right),\left(g_{4},0.4,0.5\right),\left(g_{5},0.4,0.6\right),\left(g_{6},0.4,0.7\right)\right\}\right\} \end{array}\right\},\\ \left(H_{C},E\right)=\left\{\begin{array}{c} \left\{H\left(q_{1}\right)=\left\{\left(g_{1},0.6,0.6\right),\left(g_{2},0.7,0.5\right),\left(g_{3},0.6,0.4\right),\left(g_{4},0.4,0.6\right),\left(g_{5},0.4,0.6\right),\left(g_{6},0.5,0.6\right)\right\}\right\}\\ \left\{H\left(q_{2}\right)=\left\{\left(g_{1},0.7,0.2\right),\left(g_{2},0.6,0.5\right),\left(g_{3},0.3,0.7\right),\left(g_{4},0.7,0.6\right),\left(g_{5},0.7,0.5\right),\left(g_{6},0.6,0.1\right)\right\}\right\}\\ \left\{H\left(q_{3}\right)=\left\{\left(g_{1},0.8,0.4\right),\left(g_{2},0.3,0.5\right),\left(g_{3},0.7,0.4\right),\left(g_{4},0.6,0.5\right),\left(g_{5},0.6,0.6\right),\left(g_{6},0.6,0.2\right)\right\}\right\}\\ \left\{H\left(q_{4}\right)=\left\{\left(g_{1},0.9,0.1\right),\left(g_{2},0.6,0.7\right),\left(g_{3},0.6,0.2\right),\left(g_{4},0.6,0.6\right),\left(g_{5},0.4,0.5\right),\left(g_{6},0.7,0.5\right)\right\}\right\}\\ \left\{H\left(q_{5}\right)=\left\{\left(g_{1},0.8,0.3\right),\left(g_{2},0.6,0.4\right),\left(g_{3},0.4,0.4\right),\left(g_{4},0.3,0.4\right),\left(g_{5},0.6,0.4\right),\left(g_{6},0.6,0.4\right)\right\}\right\}\\ \left\{H\left(q_{6}\right)=\left\{\left(g_{1},0.9,0.2\right),\left(g_{2},0.6,0.7\right),\left(g_{3},0.5,0.4\right),\left(g_{4},0.5,0.5\right),\left(g_{5},0.7,0.6\right),\left(g_{6},0.3,0.5\right)\right\}\right\} \end{array}\right\},\\ \left(I_{D},E\right)=\left\{\begin{array}{c} \left\{I\left(q_{1}\right)=\left\{\left(g_{1},0.3,0.7\right),\left(g_{2},0.7,0.3\right),\left(g_{3},0.7,0.4\right),\left(g_{4},0.6,0.6\right),\left(g_{5},0.6,0.4\right),\left(g_{6},0.5,0.6\right)\right\}\right\}\\ \left\{I\left(q_{2}\right)=\left\{\left(g_{1},0.8,0.4\right),\left(g_{2},0.4,0.4\right),\left(g_{3},0.5,0.7\right),\left(g_{4},0.5,0.6\right),\left(g_{5},0.6,0.5\right),\left(g_{6},0.7,0.8\right)\right\}\right\}\\ \left\{I\left(q_{3}\right)=\left\{\left(g_{1},0.7,0.3\right),\left(g_{2},0.4,0.3\right),\left(g_{3},0.5,0.4\right),\left(g_{4},0.6,0.5\right),\left(g_{5},0.7,0.6\right),\left(g_{6},0.6,0.7\right)\right\}\right\}\\ \left\{I\left(q_{4}\right)=\left\{\left(g_{1},0.9,0.1\right),\left(g_{2},0.6,0.6\right),\left(g_{3},0.4,0.7\right),\left(g_{4},0.7,0.6\right),\left(g_{5},0.5,0.5\right),\left(g_{6},0.4,0.6\right)\right\}\right\}\\ \left\{I\left(q_{5}\right)=\left\{\left(g_{1},0.8,0.3\right),\left(g_{2},0.5,0.4\right),\left(g_{3},0.6,0.6\right),\left(g_{4},0.7,0.4\right),\left(g_{5},0.8,0.6\right),\left(g_{6},0.6,0.3\right)\right\}\right\}\\ \left\{I\left(q_{6}\right)=\left\{\left(g_{1},0.6,0.1\right),\left(g_{2},0.4,0.7\right),\left(g_{3},0.6,0.5\right),\left(g_{4},0.4,0.8\right),\left(g_{5},0.5,0.6\right),\left(g_{6},0.5,0.6\right)\right\}\right\} \end{array}\right\}. \end{array}$$

These four PFSS are represented by the following PFSM, respectively:

(31) $$\begin{array}{c} A=\left[\begin{array}{cccccc} \left(0.5,0.7\right) & \left(0.8,0.2\right) & \left(0.8,0.3\right) & \left(0.9,0.1\right) & \left(0.9,0.3\right) & \left(0.8,0.4\right)\\ \left(0.8,0.5\right) & \left(0.5,0.5\right) & \left(0.4,0.5\right) & \left(0.4,0.7\right) & \left(0.6,0.5\right) & \left(0.4,0.7\right)\\ \left(0.7,0.4\right) & \left(0.6,0.7\right) & \left(0.6,0.4\right) & \left(0.6,0.7\right) & \left(0.7,0.4\right) & \left(0.6,0.4\right)\\ \left(0.7,0.6\right) & \left(0.5,0.6\right) & \left(0.5,0.5\right) & \left(0.7,0.6\right) & \left(0.5,0.4\right) & \left(0.4,0.5\right)\\ \left(0.6,0.6\right) & \left(0.5,0.5\right) & \left(0.5,0.6\right) & \left(0.5,0.5\right) & \left(0.5,0.4\right) & \left(0.5,0.6\right)\\ \left(0.5,0.7\right) & \left(0.5,0.8\right) & \left(0.4,0.7\right) & \left(0.5,0.6\right) & \left(0.6,0.3\right) & \left(0.3,0.6\right) \end{array}\right],\\ B=\left[\begin{array}{cccccc} \left(0.4,0.8\right) & \left(0.7,0.2\right) & \left(0.8,0.3\right) & \left(0.8,0.3\right) & \left(0.7,0.3\right) & \left(0.8,0.6\right)\\ \left(0.8,0.6\right) & \left(0.5,0.5\right) & \left(0.4,0.7\right) & \left(0.6,0.7\right) & \left(0.6,0.6\right) & \left(0.5,0.7\right)\\ \left(0.7,0.3\right) & \left(0.6,0.7\right) & \left(0.5,0.4\right) & \left(0.7,0.7\right) & \left(0.5,0.4\right) & \left(0.6,0.4\right)\\ \left(0.6,0.6\right) & \left(0.5,0.3\right) & \left(0.5,0.5\right) & \left(0.5,0.6\right) & \left(0.5,0.4\right) & \left(0.4,0.5\right)\\ \left(0.6,0.6\right) & \left(0.4,0.7\right) & \left(0.4,0.6\right) & \left(0.5,0.7\right) & \left(0.6,0.4\right) & \left(0.4,0.6\right)\\ \left(0.7,0.7\right) & \left(0.4,0.8\right) & \left(0.5,0.7\right) & \left(0.5,0.4\right) & \left(0.5,0.3\right) & \left(0.4,0.7\right) \end{array}\right],\\ C=\left[\begin{array}{cccccc} \left(0.6,0.6\right) & \left(0.7,0.2\right) & \left(0.8,0.4\right) & \left(0.9,0.1\right) & \left(0.8,0.3\right) & \left(0.9,0.2\right)\\ \left(0.7,0.5\right) & \left(0.6,0.5\right) & \left(0.3,0.5\right) & \left(0.6,0.7\right) & \left(0.6,0.4\right) & \left(0.6,0.7\right)\\ \left(0.6,0.4\right) & \left(0.3,0.7\right) & \left(0.7,0.4\right) & \left(0.6,0.2\right) & \left(0.4,0.4\right) & \left(0.5,0.4\right)\\ \left(0.4,0.6\right) & \left(0.7,0.6\right) & \left(0.6,0.5\right) & \left(0.6,0.6\right) & \left(0.3,0.4\right) & \left(0.5,0.5\right)\\ \left(0.4,0.6\right) & \left(0.7,0.5\right) & \left(0.6,0.6\right) & \left(0.4,0.5\right) & \left(0.6,0.4\right) & \left(0.7,0.6\right)\\ \left(0.5,0.6\right) & \left(0.6,0.1\right) & \left(0.6,0.2\right) & \left(0.7,0.5\right) & \left(0.6,0.4\right) & \left(0.3,0.5\right) \end{array}\right],\\ D=\left[\begin{array}{cccccc} \left(0.3,0.7\right) & \left(0.8,0.4\right) & \left(0.7,0.3\right) & \left(0.9,0.1\right) & \left(0.8,0.3\right) & \left(0.6,0.1\right)\\ \left(0.7,0.3\right) & \left(0.4,0.4\right) & \left(0.4,0.3\right) & \left(0.6,0.6\right) & \left(0.5,0.4\right) & \left(0.4,0.7\right)\\ \left(0.7,0.4\right) & \left(0.5,0.7\right) & \left(0.5,0.4\right) & \left(0.4,0.7\right) & \left(0.6,0.6\right) & \left(0.6,0.5\right)\\ \left(0.6,0.6\right) & \left(0.5,0.6\right) & \left(0.6,0.5\right) & \left(0.7,0.6\right) & \left(0.7,0.4\right) & \left(0.4,0.8\right)\\ \left(0.6,0.4\right) & \left(0.6,0.5\right) & \left(0.7,0.6\right) & \left(0.5,0.5\right) & \left(0.8,0.6\right) & \left(0.5,0.6\right)\\ \left(0.5,0.6\right) & \left(0.7,0.8\right) & \left(0.6,0.7\right) & \left(0.4,0.6\right) & \left(0.6,0.3\right) & \left(0.5,0.6\right) \end{array}\right]. \end{array}$$

By using score matrix definition,

(32) $$\begin{array}{c} S_{A}=\left[\begin{array}{cccccc} -0.24 & 0.6 & 0.55 & 0.8 & 0.72 & 0.48\\ 0.39 & 0 & -0.09 & -0.33 & 0.11 & -0.33\\ 0.33 & -0.13 & 0.2 & -0.13 & 0.33 & 0.2\\ 0.13 & -0.11 & 0 & 0.13 & 0.09 & -0.09\\ 0 & 0 & -0.11 & 0 & 0.09 & -0.11\\ -0.24 & -0.39 & -0.33 & -0.11 & 0.27 & -0.27 \end{array}\right],\\ S_{B\;}=\left[\begin{array}{cccccc} -0.48 & 0.45 & 0.55 & 0.55 & 0.4 & 0.28\\ 0.28 & 0 & -0.33 & -0.13 & 0 & -0.24\\ 0.4 & -0.13 & 0.09 & 0 & 0.09 & 0.2\\ 0 & 0.16 & 0 & -0.11 & 0.09 & -0.09\\ 0 & -0.13 & -0.2 & -0.24 & 0.2 & -0.2\\ 0 & -0.48 & -0.24 & 0.09 & 0.16 & -0.33 \end{array}\right],\\ S_{C}=\left[\begin{array}{cccccc} 0 & 0.45 & 0.48 & 0.8 & 0.55 & 0.77\\ 0.24 & 0.11 & -0.16 & -0.13 & 0.2 & -0.13\\ 0.2 & -0.4 & 0.33 & 0.32 & 0 & 0.09\\ -0.2 & 0.13 & 0.11 & 0 & -0.07 & 0\\ 0.2 & 0.24 & 0 & -0.09 & 0.2 & 0.13\\ -0.11 & 0.35 & 0.32 & 0.24 & 0,2 & -0.16 \end{array}\right],\\ S_{D}=\left[\begin{array}{cccccc} -0,4 & -0.48 & 0.4 & 0.8 & 0.55 & 0.35\\ 0.4 & 0 & 0.07 & 0 & 0.09 & -0.33\\ 0.33 & -0.24 & 0.09 & -0.33 & 0 & 0.11\\ 0 & -0.11 & 0.11 & 0.13 & 0.33 & -0.48\\ 0.2 & 0.11 & 0.13 & 0 & 0.28 & -0.11\\ -0.11 & -0.15 & -0.13 & -0.2 & 0.27 & -0.11 \end{array}\right]. \end{array}$$

By using the definition of utility matrix,

(33) $$\begin{array}{c} S_{\left(A,B,C,D\right)}=\left[\begin{array}{cccccc} 0.64 & 0.18 & -0.88 & -1.35 & -0.78 & -0.92\\ -0.53 & -0.11 & 0.01 & -0.7 & -0.18 & 0.37\\ -0.6 & 0.64 & -0.31 & -0.12 & 0.24 & -0.2\\ 0.33 & -0.29 & -0.22 & 0.11 & -0.26 & 0.48\\ -0.4 & -0.22 & -0.04 & 0.33 & -0.59 & 0.07\\ -0.02 & -0.11 & -0.28 & -0.24 & -0.36 & 0.33 \end{array}\right]. \end{array}$$

Now,

(34) $$\begin{array}{c} \text{total score}=\begin{array}{c} g_{1}\\ g_{2}\\ g_{3}\\ g_{4}\\ g_{5}\\ g_{6} \end{array}\left[\begin{array}{c} 3.11\\ 1.21\\ 0.35\\ 0.15\\ 0.85\\ 0.68 \end{array}\right]. \end{array}$$

From the above results *x*_1_, the 1st brand having maximum value, in this fashion, we conclude from the judgment of four experts that Honda is the best brand for business.

Ranking of alternative *g*_1_ > *g*_2_ > *g*_5_ > *g*_6_ > *g*_3_ > *g*_4_.

## 5. Discussion and Comparative Studies

For comparative analysis, we made the comparison of our proposed method with different methods by using their algorithm. Through this process, we can easily show the credibility of our proposed method.

### 5.1. Comparative Studies

Here, we use the method of Rathika and Subramanian [40], in which firstly authors find the complement matrices and apply the algebraic operation to compute matrices values that help to derive the scoring matrix, and after this, the authors compute total score matrix belonging to individual alternative by using score matrix. Score function remains a matrix that corresponds to all the characteristics of a real matrix. Finally, using the rank of the alternatives, build the total score matrix to choose the best alternative. The above process can also be presented as follows.

#### 5.1.1. Algorithm (see [40])

  Step 1: input IFSSs and obtain intuitionistic fuzzy soft matrices (IFSM)
  Step 2: take complement of IFSS and obtain complement IFSM from these sets
  Step 3: find (*A* − *B* − *C* − *D*), (*A*^*c*^ − *B*^*c*^ − *C*^*c*^ − *D*^*c*^), and value matrix of these IFSMs
  Step 4: compute the score of IFSMs and complement IFSMs
  Step 5: calculate the total score *S*_*i*_ for *x*_*i*_ in-universe
  Step 6: we conclude that element *x*_*i*_ of the total score matrix *S*_*i*_ has maximum value which is the best alternative

If max *S*_*i*_ has more values besides one value, then rerun the development by revising the parameter.

Value matrix

(35) $$\begin{array}{c} V\left(A-B-C-D\right)=\left[\begin{array}{cccccc} -0.4 & 0,3 & 0.3 & 0.5 & 0.4 & 0\\ 0.4 & -0.1 & -0.4 & -0.3 & -0.1 & -0.3\\ 0.2 & -0.4 & 0.1 & -0.3 & -0.2 & 0\\ -0.2 & -0.1 & 0 & -0.1 & 0.1 & -0.4\\ -0.2 & -0.3 & -0.2 & -0.3 & -0.1 & -0.2\\ -0.2 & -0.4 & -0.3 & -0.2 & 0.1 & -0.4 \end{array}\right],\\ V\left(A^{C}-B^{C}-C^{C}-D^{C}\right)=\left[\begin{array}{cccccc} 0 & -0.6 & -0.5 & -0.8 & -0.6 & -0.8\\ -0.5 & -0.2 & -0.4 & 0 & -0.2 & 0.1\\ -0.4 & 0.1 & -0.3 & -0.4 & -0.3 & -0.2\\ -0.1 & -0.4 & -0,1 & -0.5 & -0.3 & 0\\ -0.2 & -0.2 & -0.1 & -0.1 & -0.4 & -0.1\\ -0.1 & -0.6 & -0.4 & -0.3 & -0.3 & 0 \end{array}\right]. \end{array}$$

Now, calculate the score matrix

(36) $$\begin{array}{c} S=\left[\begin{array}{cccccc} -0.4 & 0.9 & 0.8 & 1.3 & 1.0 & 0.8\\ 0.9 & 0.1 & 0 & -0.3 & 0.1 & -0.4\\ 0.6 & -0.3 & 0.4 & 0.1 & 0.1 & 0.2\\ -0.1 & 0 & 0.2 & 0.4 & 0.4 & -0.4\\ 0 & -0.1 & -0.1 & -0.2 & 0.3 & -0.1\\ -0.1 & 0.2 & 0.1 & 0.1 & 0.4 & -0.4 \end{array}\right],\\ \text{total score}=\begin{array}{c} g_{1}\\ g_{2}\\ g_{3}\\ g_{4}\\ g_{5}\\ g_{6} \end{array}\left[\begin{array}{c} 4.4\\ 0.4\\ 1.1\\ 0.5\\ 0.2\\ 0.3 \end{array}\right]. \end{array}$$

Ranking of alternative *g*_1_ > *g*_3_ > *g*_4_ > *g*_2_ > *g*_6_ > *g*_5_.

Now, we use the method of Rajarajeswari and Dhanalakshmi [29], in which firstly authors find the complement matrices and apply the algebraic operation to compute matrices values that help to derive the scoring matrix, and after this, the authors compute total score belonging to individually alternative by using score matrix. Score function remains a matrix that corresponds to all the characteristics of a real matrix, finally using the rank of the alternatives to build the total score matrix to choose the best alternative. The above process can also be presented is as follows.

#### 5.1.2. Algorithm (see [29])

  Step 1: input IFSSs and obtain IFSMs
  Step 2: take complement of IFSS and obtain complement IFSM from these sets
  Step 3: find (*A*+*B*+*C*+*D*), (*A*^*c*^+*B*^*c*^+*C*^*c*^+*D*^*c*^), and value matrix of these IFSMs
  Step 4: compute the score of IFSMs and complement IFSMs
  Step 5: calculate the total score *S*_*i*_ for *x*_*i*_ in-universe
  Step 6: we conclude that element *x*_*i*_ of the total score matrix *S*_*i*_ has maximum value which is the best alternative

If max *S*_*i*_ has more values besides one value, then rerun the development by revising the parameter.

Value matrix

(37) $$\begin{array}{c} V\left(A+B+C+D\right)=\left[\begin{array}{cccccc} 0.0 & 0.6 & 0.5 & 0.8 & 0.6 & 0.8\\ 0.5 & 0.2 & 0.1 & 0.0 & 0.2 & -0.1\\ 0.4 & -0.1 & 0.3 & 0.5 & 0.3 & 0.2\\ 0.1 & 0.4 & 0.1 & 0.1 & 0.3 & 0.2\\ 0.2 & 0.2 & 0.1 & 0.0 & 0.4 & 0.1\\ 0.1 & 0.6 & 0.4 & 0.3 & 0.3 & 0.0 \end{array}\right],\\ V\left(A^{C}+B^{C}+C^{C}+D^{C}\right)=\left[\begin{array}{cccccc} 0.5 & -0.3 & -0.3 & -0.5 & -0.4 & 0.0\\ -0.1 & 0.1 & 0.4 & 0.3 & 0.1 & 0.3\\ 0.0 & 0.4 & -0.1 & 0.3 & 0.2 & 0.0\\ 0.2 & 0.1 & 0.0 & 0.1 & 0.1 & 0.4\\ 0.2 & 0.3 & 0.2 & 0.3 & 0.1 & 0.2\\ 0.2 & 0.4 & 0.3 & 0.2 & -0.1 & 0.4 \end{array}\right]. \end{array}$$

Now, calculate the score matrix

(38) $$\begin{array}{c} S=\left[\begin{array}{cccccc} -0.5 & 0.9 & 0.8 & 1.3 & 1.0 & 0.8\\ 0.6 & 0.1 & -0.3 & -0.3 & 0.1 & -0.4\\ 0.4 & -0.5 & 0.4 & 0.2 & 0.1 & 0.2\\ -0.1 & 0.3 & 0.1 & 0.0 & 0.2 & -0.2\\ 0.0 & -0.1 & -0.1 & -0.3 & 0.3 & -0.1\\ -0.1 & 0.2 & 0.1 & 0.1 & 0.4 & -0.4 \end{array}\right],\\ \text{total score}=\begin{array}{c} g_{1}\\ g_{2}\\ g_{3}\\ g_{4}\\ g_{5}\\ g_{6} \end{array}\left[\begin{array}{c} 4.3\\ -0.2\\ 0.8\\ 0.3\\ -0.3\\ 0.3 \end{array}\right]. \end{array}$$

Ranking of alternative *g*_1_ > *g*_3_ > *g*_4_ > *g*_6_ > *g*_2_ > *g*_5_.

Now, we use the method of Borah et al. [41], in which firstly authors select the parameter and convert them into matrices form. Secondly, apply the algebraic operation to compute matrices values that help to derive the optimum matrix, finally using the rank of the alternatives to build the optimum matrix to choose the best alternative. The above process can also be presented as follows.

#### 5.1.3. Algorithm [41]

  Step 1: select a set of parameters
  Step 2: obtain fuzzy soft matrices (IFSS) from these sets
  Step 3: evaluate the cross-product of FSM
  Step 4: enumerate the optimum subscript matrix
  Step 5: compute the best alternative that is having max value

We have

(39) $$\begin{array}{c} A\times B\times C\times D=\begin{array}{c} g_{1}\\ g_{2}\\ g_{3}\\ g_{4}\\ g_{5}\\ g_{6} \end{array}\left[\begin{array}{c} 0.036+0.3136+0.3584+0.5832+0.4032+0.3456\\ 0.3136+0.06+0.0192+0.0864+0.108+0.048\\ 0.2058+0.054+0.105+0.1008+0.084+0.108\\ 0.1008+0.0875+0.09+0.147+0.0525+0.032\\ 0.0864+0.084+0.084+0.05+0.144+0.07\\ 0.0875+0.084+0.072+0.07+0.108+0.018 \end{array}\right],\\ A\times B\times C\times D=\begin{array}{c} g_{1}\\ g_{2}\\ g_{3}\\ g_{4}\\ g_{5}\\ g_{6} \end{array}\left[\begin{array}{c} 2.04\\ 0.6352\\ 0.6576\\ 0.5098\\ 0.5184\\ 0.4395 \end{array}\right]. \end{array}$$

Ranking of alternative *g*_1_ > *g*_3_ > *g*_2_ > *g*_5_ > *g*_4_ > *g*_6_.

Furthermore, we use the algorithm of Rathika et al. [42], in which firstly authors find the complement matrices and apply the algebraic operation to compute matrices values that help to derive the scoring matrix, and after this, the authors compute total score along with individually alternative by using score matrix. Score function remains a matrix that corresponds to all the characteristics of a real matrix, finally using the rank of alternatives to build the total score matrix to choose the best alternative. The above process can also be presented is as follows.

#### 5.1.4. Algorithm (see [42])

  Step 1: input IFSSs and obtain IFSMs
  Step 2: take complement of IFSS and obtain complement IFSM from these sets
  Step 3: find (*A* − *B* − *C* − *D*), (*A*^*c*^ − *B*^*c*^ − *C*^*c*^ − *D*^*c*^), and value matrix of these IFSMs
  Step 4: compute the score of IFSMs and complement IFSMs
  Step 5: calculate the total score *S*_*i*_ for *x*_*i*_ in-universe
  Step 6: we conclude that element *x*_*i*_ of the total score matrix *S*_*i*_ has maximum value which is the best alternative

If max *S*_*i*_ has more values besides one value, then rerun the development by repeating the parameter.

(40) $$\begin{array}{c} MV\left(A-B-C-D\right)=\left[\begin{array}{cccccc} 0.3 & 0.7 & 0.7 & 0.8 & 0.7 & 0.6\\ 0.7 & 0.4 & 0.3 & 0.4 & 0.5 & 0.4\\ 0.6 & 0.3 & 0.5 & 0.4 & 0.4 & 0.5\\ 0.4 & 0.5 & 0.5 & 0.5 & 0.3 & 0.4\\ 0.4 & 0.4 & 0.4 & 0.4 & 0.5 & 0.4\\ 0.5 & 0.4 & 0.4 & 0.4 & 0.5 & 0.3 \end{array}\right],\\ MV\left(A^{o}-B^{o}-C^{o}-D^{o}\right)=\left[\begin{array}{cccccc} 0.4 & 0.2 & 0.2 & 0.1 & 0.1 & 0.1\\ 0.2 & 0.4 & 0.6 & 0.4 & 0.4 & 0.4\\ 0.3 & 0.4 & 0.3 & 0.3 & 0.3 & 0.4\\ 0.3 & 0.3 & 0.4 & 0.3 & 0.3 & 0.5\\ 0.4 & 0.3 & 0.3 & 0.5 & 0.2 & 0.3\\ 0.3 & 0.3 & 0.4 & 0.3 & 0.4 & 0.5 \end{array}\right],\\ S=\left[\begin{array}{cccccc} -0.1 & 0.5 & 0.5 & 0.7 & 0.6 & 0.5\\ 0.5 & 0.0 & -0.3 & 0.0 & 0.1 & 0.0\\ 0.3 & -0.1 & 0.2 & 0.1 & 0.1 & 0.1\\ 0.1 & 0.2 & 0.1 & 0.2 & 0.0 & -0.1\\ 0.0 & 0.1 & -0.1 & -0.1 & 0.3 & 0.1\\ 0.2 & 0.1 & 0.0 & 0.1 & 0.1 & -0.2 \end{array}\right],\\ \text{total score}=\begin{array}{c} g_{1}\\ g_{2}\\ g_{3}\\ g_{4}\\ g_{5}\\ g_{6} \end{array}\left[\begin{array}{c} 2.7\\ 0.3\\ 0.7\\ 0.5\\ 0.3\\ 0.3 \end{array}\right]. \end{array}$$

Ranking of alternative *g*_1_ > *g*_3_ > *g*_4_ > *g*_2_ > *g*_5_ > *g*_6_.

Also, we use the technique of Chetia and Das [43], in which firstly authors find the union matrices and apply the algebraic operation to compute matrices values that help to derive the weight matrix, finally using the rank of the alternatives to build the weight matrix to choose the best alternative. The above process can also be presented as follows.

#### 5.1.5. Algorithm (see [43])

Step 1: select the FSS set of parameters
Step 2: obtain FSMs from these sets
Step 3: find the union of these FSMs
Step 4: enumerate the weight along with the individual item by taking rowwise sum membership values
Step 5: compute the best alternative that is having max value
  (41) $$\begin{array}{c} A\cup B\cup C\cup D=\left[\begin{array}{cccccc} \left(0.6,\;0.6\right) & \left(0.8,\;0.2\right) & \left(0.8,\;0.3\right) & \left(0.9,\;0.1\right) & \left(0.9,\;0.3\right) & \left(0.9,\;0.1\right)\\ \left(0.8,\;0.3\right) & \left(0.6,\;0.4\right) & \left(0.4,\;0.3\right) & \left(0.6,\;0.6\right) & \left(0.6,\;0.4\right) & \left(0.6,\;0.7\right)\\ \left(0.7,\;0.3\right) & \left(0.6,\;0.7\right) & \left(0.7,\;0.4\right) & \left(0.7,\;0.2\right) & \left(0.7,\;0.4\right) & \left(0.6,\;0.4\right)\\ \left(0.7,\;0.6\right) & \left(0.7,\;0.3\right) & \left(0.6,\;0.5\right) & \left(0.7,\;0.6\right) & \left(0.7,\;0.4\right) & \left(0.7,\;0.5\right)\\ \left(0.6,\;0.4\right) & \left(0.7,\;0.5\right) & \left(0.7,\;0.6\right) & \left(0.5,\;0.5\right) & \left(0.8,\;0.4\right) & \left(0.7,\;0.6\right)\\ \left(0.7,\;0.6\right) & \left(0.7,\;0.1\right) & \left(0.6,\;0.2\right) & \left(0.7,\;0.4\right) & \left(0.6,\;0.3\right) & \left(0.5,\;0.5\right) \end{array}\right]. \end{array}$$

Now, weights matrix of the bikes of different brands,

(42) $$\begin{array}{c} W=\begin{array}{c} g_{1}\\ g_{2}\\ g_{3}\\ g_{4}\\ g_{5}\\ g_{6} \end{array}\left[\begin{array}{c} 0.6+0.8+0.8+0.9+0.9+0.9\\ 0.8+0.6+0.4+0.6+0.6+0.6\\ 0.7+0.6+0.7+0.7+0.7+0.6\\ 0.7+0.7+0.6+0.7+0.7+0.7\\ 0.6+0.7+0.7+0.5+0.8+0.7\\ 0.7+0.7+0.6+0.7+0.6+0.5 \end{array}\right],\\ W=\begin{array}{c} g_{1}\\ g_{2}\\ g_{3}\\ g_{4}\\ g_{5}\\ g_{6} \end{array}\left[\begin{array}{c} 4.9\\ 3.6\\ 4.0\\ 4.1\\ 4.0\\ 3.8 \end{array}\right]. \end{array}$$

Ranking of alternative *g*_1_ > *g*_4_ > *g*_3_=*g*_5_ > *g*_6_ > *g*_2_.

The results obtained through existing methodologies with the proposed technique with their score values are given in Table 1.

### 5.2. Exploration by Comparison

With the ongoing research and comparison, the results obtained by our proposed method and existing procedures are in Table 1. However, concerning available decision-making strategies, the leading advantage along with this proposed mechanism is that it contains extra details using MD, nonmembership, along with the unreliability of a small number of features supposed to briefing data unreliability. It is an important appliance for resolving imprecise and inaccurate figures of decision-making procedures. Consequently, the promotion of score value associated with individual parameters should not influence the additional parameters; for this reason, the unpredictable mislaying of facts will take place in the procedure. The advantage of an organized approach and the accompanying measures and current approaches is not only that recognizes the level of prejudice, but the level of resemblance in the middle of what is seen as negative factors. It is most appropriate technique to integrate uncertain and vague data in decision-making procedure. Analyze obtained results along with the various other researchers' work on the score-matrix problem by using different operators; our developed proposed method is fully rational with different available approaches. It is compiled in Table 1 and references. We made a comparative analysis by taking similar data in the decision-making of all mentioned approaches. In the future, our developed PFSM can be used for a variety of purposes by its nature of group decision-making, data revival, pattern perception, size contraction, and data drilling.

## 6. Conclusion

In this paper, we developed some logical operators such as OR-operation and AND-operation for PFSS with their fundamental characteristics. Also, some novel operations such as necessity and possibility operations have been presented with their fundamental properties. A decision-making approach has been constructed for PFSM based on a score matrix and utility matrix. To confirm the validity of our established approach, a comprehensive numerical example has been developed. To express the legitimacy, effectiveness, and efficiency of our proposed method, a logical comparison between the current work and the proposed approach is also provided. The obtained consequences show that the developed technique is more reliable compared to existing techniques. Future research will focus on offering several other operators under the PFSS environment to address decision-making issues. Many other structures such as topological, algebra, and orderly structures can be developed and discussed in the environment under consideration. The proposed idea can be applied to many issues in real life, including the medical profession, pattern recognition, and economics.

## Figures and Tables

**Table 1:** Comparative analysis with existing operators.

	*g*_1_	*g*_2_	*g*_3_	*g*_4_	*g*_5_	*g*_6_	Alternatives ranking
Rathika and Subramanian [40]	4.4	0.4	1.1	0.5	0.2	0.3	*g*_1_ > *g*_3_ > *g*_4_ > *g*_2_ > *g*_6_ > *g*_5_
Rajarajeswari and Dhanalakshmi [29]	4.3	−0.2	0.8	0.3	−0.3	0.3	*g*_1_ > *g*_3_ > *g*_4_=*g*_6_ > *g*_2_ > *g*_5_
Borah et al. [41]	2.04	0.64	0.66	0.51	0.52	0.44	*g*_1_ > *g*_3_ > *g*_2_ > *g*_5_ > *g*_4_ > *g*_6_
Rathika et al. [42]	2.7	0.3	0.7	0.5	0.3	0.3	*g*_1_ > *g*_3_ > *g*_4_ > *g*_2_=*g*_5_=*g*_6_
Chetia and Das [43]	4.9	3.6	4.0	4.1	4.0	3.8	*g*_1_ > *g*_4_ > *g*_3_=*g*_5_ > *g*_6_ > *g*_2_
Proposed approach	3.11	1.21	0.35	0.15	0.85	0.68	*g*_1_ > *g*_2_ > *g*_5_ > *g*_6_ > *g*_3_ > *g*_4_

## Data Availability

No data were used in this article.
