# Corrigendum to “A Decision-Making Approach Based on Score Matrix for Pythagorean Fuzzy Soft Set”

**DOI:** 10.1155/2022/9845825

**Published:** 2022-11-18

**Authors:** Imran Siddique, Rana Muhammad Zulqarnain, Rifaqat Ali, Alhanouf Alburaikan, Aiyared Iampan, Hamiden Abd El-Wahed Khalifa

**Affiliations:** ^1^Department of Mathematics, School of Science, University of Management and Technology, Lahore 54770, Pakistan; ^2^Department of Mathematics, School of Science, Sialkot Campus, University of Management and Technology, Lahore, Pakistan; ^3^Department of Mathematics, College of Science and Arts, King Khalid University, Abha, Muhayil 61413, Saudi Arabia; ^4^Department of Mathematics, College of Science and Arts, Al-Badaya, Qassim University, Buraydah, Saudi Arabia; ^5^Department of Mathematics, School of Science, University of Phayao, Mae Ka, Mueang, Phayao 56000, Thailand; ^6^Operations Research Department, Faculty of Graduate Studies for Statistical Research, Cairo University, Giza, Egypt

In the article titled “A Decision-Making Approach Based on Score Matrix for Pythagorean Fuzzy Soft Set” [1], there was an error in the fourth listed affiliation. This is corrected as shown above.
